# Spexin mRNA profile and its response to different photoperiods in Chinese Yangzhou geese (*Anas cygnoides*)

**DOI:** 10.3389/fvets.2022.961431

**Published:** 2022-09-02

**Authors:** Jie Liu, Shudi Dai, Xibing Shao, Chuankun Wei, Zichun Dai, Pengxia Yang, Mingming Lei, Rong Chen, Huanxi Zhu

**Affiliations:** ^1^Key Laboratory of Crop and Livestock Integration, Ministry of Agriculture, Nanjing, China; ^2^Institute of Animal Science, Jiangsu Academy of Agricultural Sciences, Nanjing, China; ^3^School of Life Science, Jiangsu University, Zhenjiang, China; ^4^Anhui Tianzhi-jiao Goose Industry Co., Ltd., Chuzhou, China

**Keywords:** goose, spexin, GalR2/3, gonadotrope-axis, photoperiods

## Abstract

Spexin (SPX, NPQ), a novel neuropeptide composed of 14 amino acid residues, is evolutionarily conserved among different species. Spexin has been suggested to have pleiotropic functions in mammals. However, reports on spexin in birds are limited. To clarify the role of spexin in goose reproduction, the spexin gene was cloned and analyzed. Analysis of tissue distribution by RT-PCR showed that the expression of spexin and its two receptors was widespread. During the long photoperiod, the expression levels of spexin in the pituitary and hypothalamus and of GALR2/3 in the pituitary decreased, and the GnRH, LHβ, and FSHβ expression levels increased significantly. This suggests that a long photoperiod regulates reproductive activities by activating the gonadotrope-axis, which is modulated by decreased spexin levels.

## Introduction

Spexin is a highly conserved peptide recently discovered using bioinformatics methods based on hidden Markov model screening (1). It is evolutionarily conserved across taxa, from fish to mammals, with an amino acid length of 14 (2, 3). Spexin mRNA and proteins are widely distributed in the central nervous system and peripheral tissues of various species (4), and gene structural analysis shows that it belongs to the spexin/kisspeptin/galanin gene family (5). Moreover, spexin has been shown to be a natural ligand for galanin receptor 2/3 (GALR2/3) (5) and plays an important role in feeding behavior, gastrointestinal motility, obesity, diabetes, energy metabolism, endocrine and mental diseases, and cardiovascular function (3).

Despite the growing knowledge on spexin in many animal species, there is little research focusing on birds. Farzin et al. (6) found that spexin affects the regulation of central food intake and nutritional behavior in broiler chickens, and Kołodziejski et al. (7) found that spexin is involved in the regulation of their metabolism. However, there is neither a description of the sequence and distribution of spexin in geese nor the impact of spexin on goose reproduction. Therefore, we investigated the characteristics and tissue distribution of spexin in geese. In addition, studies on the reproductive effects of spexin have mainly been carried out in teleosts, and it has been suggested that spexin may play a role at the level of the hypothalamus and pituitary. Moreover, the results in teleosts have not been consistent, and consequently the role of spexin in reproductive activities is unclear (8).

In our previous research, we found that artificial photoperiod can regulate the egg laying of Yangzhou geese (9). In addition, the preliminary study found that the initiation of egg laying activity of Yangzhou geese by prolonged light may be mediated by higher FSHβ and LHβ (9). Studies on fish have shown that spexin may be involved in the regulation of FSHβ and LHβ expression (10, 11), but it is not clear whether the effect of photoperiod on FSHβ and LHβ is mediated by spexin. Studies on different animal models have pointed out that photoperiod may regulate animal reproductive behavior by changing the levels of GnIH (12, 13), thyrotropin (14, 15), kisspeptin (13) and genomic methylation (16). However, there is no report on the correlation between spexin and photoperiod in birds. Here, we try to use the mature photoperiod model, which regulates egg laying (9), to explore the role of spexin in goose reproductive activities under different photoperiods.

## Materials and methods

### Ethical approval

The experimental procedures were approved by the Research Committee of Jiangsu Academy of Agricultural Sciences and conducted with adherence to the Regulations for the Administration of Affairs Concerning Experimental Animals (Decree No. 63 of the Jiangsu Academy of Agricultural Science on July 8, 2014).

### Animals and experiment procedure

Yangzhou geese of the same batch and source aged 130 days were selected as the research object, and the male female ratio was 1:5. All experimental geese were operated according to the anti-seasonal laying light treatment procedure. They had been treated with light for 40 days according to the light treatment procedure of L: D = 19:5 h at the age of 90 days. They were fed in combination with the limited feeding of reserve goose feed and sent to the poultry farm of the experimental animal base of Zhuzhen town Academy of Agricultural Sciences (118°62′E, 32°48′N), Luhe District, Nanjing at the age of 130 days. On February 15, 2021, the test geese were treated according to the light program of L: D = 7 h: 17 h for 60 days at the age of 130 days, to remove the light passivation effect of breeding geese, and gradually implement the free feeding of reserve breeding geese at the age of 160 days to restore their physical condition. The experimental geese began to adopt the light program of breeding period with L: D = 12 h: 12 h at the age of 190 days, combined with the feed of breeding geese during egg laying period, and laid eggs for the first time on May 12, 2021. Male and female goose samples were collected on April 9 and May 26 for follow-up analysis. The samples from April 9 were selected for mRNA tissue distribution analysis.

### Total RNA isolation and cDNA synthesis

Total RNA from different tissues was extracted using TRIzol reagent (Vazyme) following the manufacturer's instructions. RNA quality was assessed by electrophoresis on a 1.2% agarose gel, and the total RNA concentration was determined by using NanoDrop 2000. The following reverse-transcribed using HiScript Q RT SuperMix for qPCR (Vazyme), according to the manufacturer's protocol.

### Gene cloning of full-length spexin and sequence analysis

Gene cloning of full-length spexin cDNA and sequence analysis Fragments (5′ and 3′) of spexin were obtained using the 5′ and 3′ RACE System for Rapid Amplification of cDNA Ends amplification kit (Invitrogen), according to the manufacturer's instructions. The specific RACE-PCR primers designed using the spexin genes sequences and Primer 3 software (http://frodo.wi.mit.edu/primer3/) are listed in Table 1.

**Table 1:** Primer sequences used in this study.

**Primers name**	**Sequences (5^′^-3^′^)**
**RACE**	
Ac-spexin-5′RACE-R1	caagtagccagggtgatcaccattttct
Ac-spexin-5RACE′-R2	tttcttcttcaacttgttcttgggctttctg
Ac-spexin-3′RACE-F	cagatgagagccagcaaaaggatctg
**Specific primers for two cDNA**	
Ac-spexin cDNA1-F	ggactccgtaaactgacagc
Ac-spexin cDNA1-R	tccaggagaaagatatgaaggac
Ac-spexin cDNA2-F	aggtccctttagactccccc
Ac-spexin cDNA2-R	tagcctggggagtccagttt
**Primers for real-time PCR to detect the expression level**	
spexin-F	gaatgcagcttgaaacacgc
spexin-R	tatccgtcaagtagccaggg
GAPDH-F	gccatcacagccacacaga
GAPDH-R	tttccccacagccttagca
GnRH-F	ctgggacccttgctgttttg
GnRH-R	aggggacttccaaccatcac
GnIH-F	atctacctaggcatgctccaa
GnIH-R	acaggcagtgacttcccaaat
LHβ-F	ccataaacgtaacggtggcg
LHβ-R	cccaaagggctgcgatacac
FSHβ-F	cctagccattgctgtgcattt
FSHβ-R	tgccaggttgctcatcaagg

Homology searches for nucleotide and amino acid sequence similarities were conducted with BLAST programs (https://blast.ncbi.nlm.nih.gov/Blast.cgi). The deduced amino acid sequence was analyzed with the Expert Protein Analysis System (http://www.expasy.org/). Domain predictions were conducted with the Simple Modular Architecture Research Tool (http://smart.emblheidelberg.de/). Multiple sequence alignment was performed using ClustalW2 (http://www.ebi.ac.uk/Tools/msa/clustalw2/). A cladogram was constructed based on amino sequence alignment by the neighbor-joining algorithm embedded in the MEGA 5 program.

### Real-time PCR analysis of mRNA expression

The synthesized cDNA was used for quantitative real-time PCR after 10 times dilution. The gene encoding GAPDH was used as a reference gene. All the primer are designed by Primer 3 software and the primer sequences shown in Table 1. The primers were synthesized by Tsingke Biotechnology Co., Ltd. The real-time PCR results were analyzed using the 2^−ΔΔCt^ method, and the abundance of mRNA was expressed as the fold change relative to the mean value of the female group with short photoperiod.

### Statistical analysis

Differences between the short and long photoperiod in female or male were analyzed by *T*-test performed using SPSS software (Version 23). The final presentation of data depends on GraphPad Prism software (9.0.0), and Adobe Illustrator (2020) software is used for picture integration in mRNA distribution results (Figure 3). Differences between treatment means were compared by the mean ± SEM (*n* = 3). The differences were considered statistically significant when *P* < 0.05.

## Results

### Sequence characteristics of spexin

**Figure 1:**
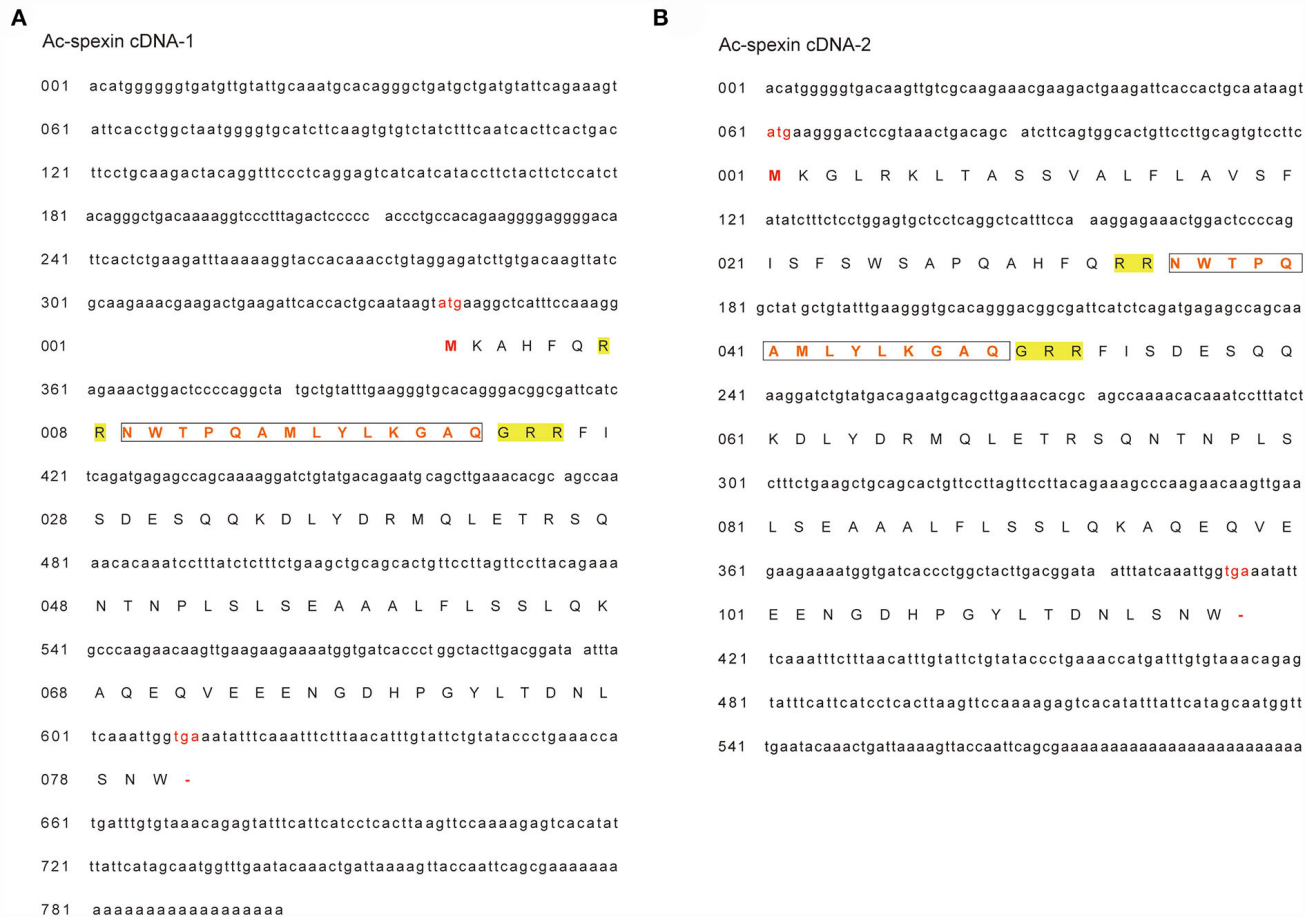
Complete cDNA sequences and deduced amino acid sequences of Anas cygnoides spexin. **(A)** Ac-spexin cDNA-1. **(B)** Ac-spexin cDNA-2. Start codon and stop codons are shown in red. The mature peptides are boxed. The predicted pro-peptide cleavage sites are in yellow.

Two cDNA sequences of spexin were found in geese by 3' and 5' RACE, and the lengths of the cDNA sequences were 600-bp (Ac-spexin cDNA-1) and 798-bp (Ac-spexin cDNA-2), respectively (Figure 1). The long spexin cDNA fragment included the open reading frame, encoding a predicted pro-peptide of 90 amino acid residues that contained a mature peptide (NWTPQAMLYLKGTQ). In contrast, the short spexin cDNA fragment encoded a predicted pro-peptide of 117 amino acid residues, including the 14-aa putative mature peptide. In addition, compared to the predicted sequence reported by NCBI (XP_013040020.1), the similarity of the Ac-spexin cDNA-1 sequence was higher. However, both the predicted pro-peptides contained cleavage sites. Interestingly, by designing specific primers for the two cDNA sequences, we found that long cDNA exists, but the relative expression is very low (or not shown). Therefore, we performed a follow-up analysis of short spexin.

### Phylogenetic analysis

**Figure 2:**
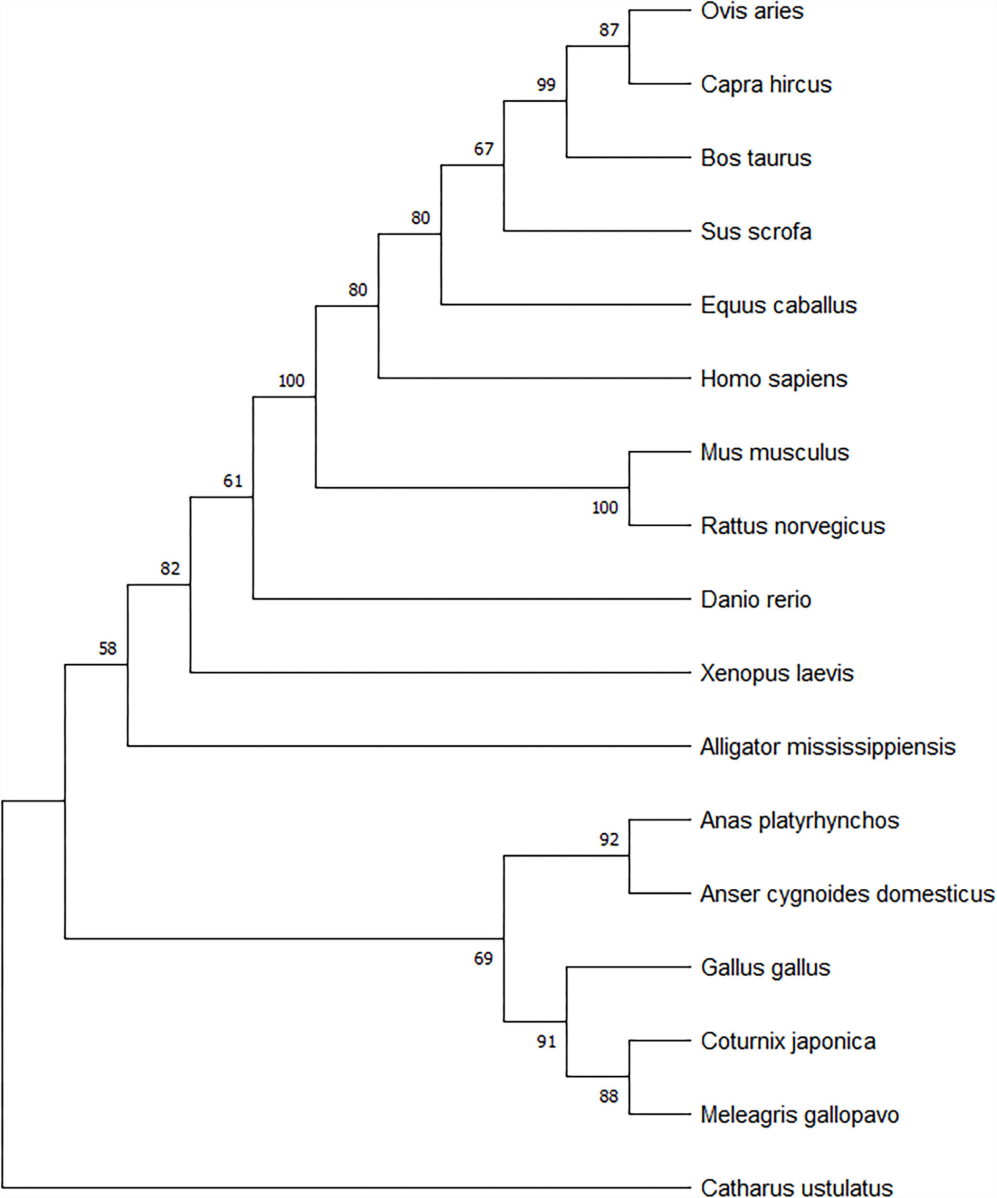
Spexin phylogenetic tree based on Neighbor-Joining method using MEGA5. The branch length scale in terms of genetic distance is indicated above the tree.

The amino acid sequences of the geese (117-aa) and other selected species were used to construct a phylogenetic tree. Phylogenetic analysis confirmed the close relationship between the swan goose (*Anser cygnoides domesticus*) and the mallard (*A. platyrhynchos*). The spexin of geese is closely related to the spexin sequences of *Gallus gallus, C. japonica*, and *M. gallopavo*. However, there were obvious species differences between birds, mammals, and fish (Figure 2).

### Tissue distribution of spexin and GALR2/3

We used real-time PCR to analyze the distribution of spexin and its two receptors (GALR2/3) in male and female geese. The distribution in male and female individuals was not significantly different.

**Figure 3:**
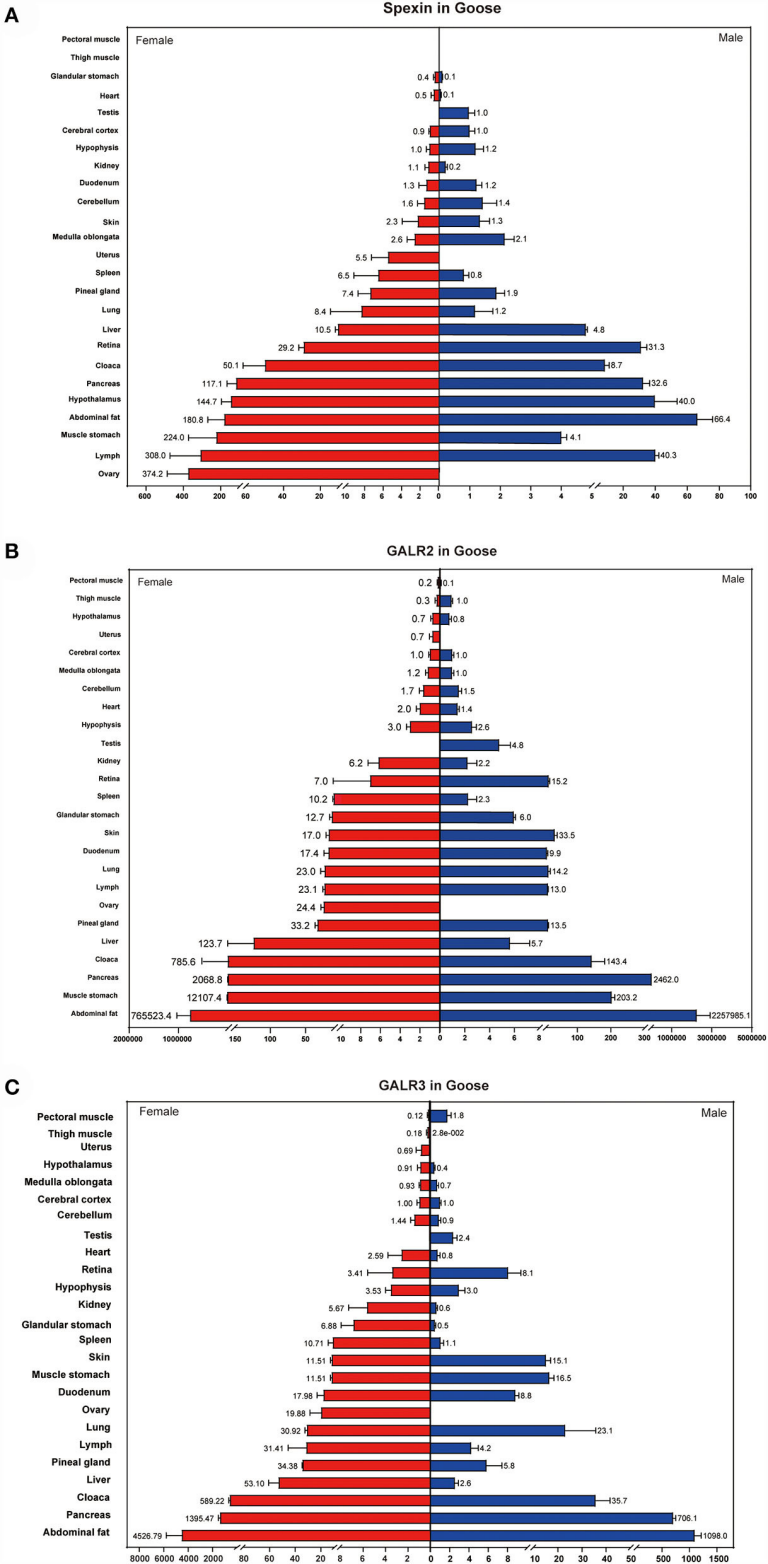
Tissue distribution of spexin and GALR2/3 in female and male goose. **(A)** Tissue distribution of spexin in female and male goose. **(B,C)** Tissue distribution of GALR2/3 in female and male goose. The expression in cerebral cortex were used as a reference. Data are expressed as mean ± SEM (*n* = 3).

The results showed that spexin transcripts were widely expressed, including in the glandular stomach, heart, testis, cerebral cortex, hypothalamus, kidney, duodenum, cerebellum, skin, medulla oblongata, uterus, spleen, pineal gland, lung, liver, retiba, cloaca, pancreas, hypophysis, abdominal fat, muscle stomach, lymph, and ovary. The expression level of spexin was higher in the ovary, lymph, stomach muscle, abdominal fat, pituitary gland, and pancreas (Figure 3A).

GALR2 and GALR3 were detected in all selected tissues. In addition, it is noteworthy that the expression of these two receptors in adipose tissue was significantly higher than that in other tissues (Figures 3B,C).

### Spexin and GALR2/3 expression during different photoperiods

**Figure 4:**
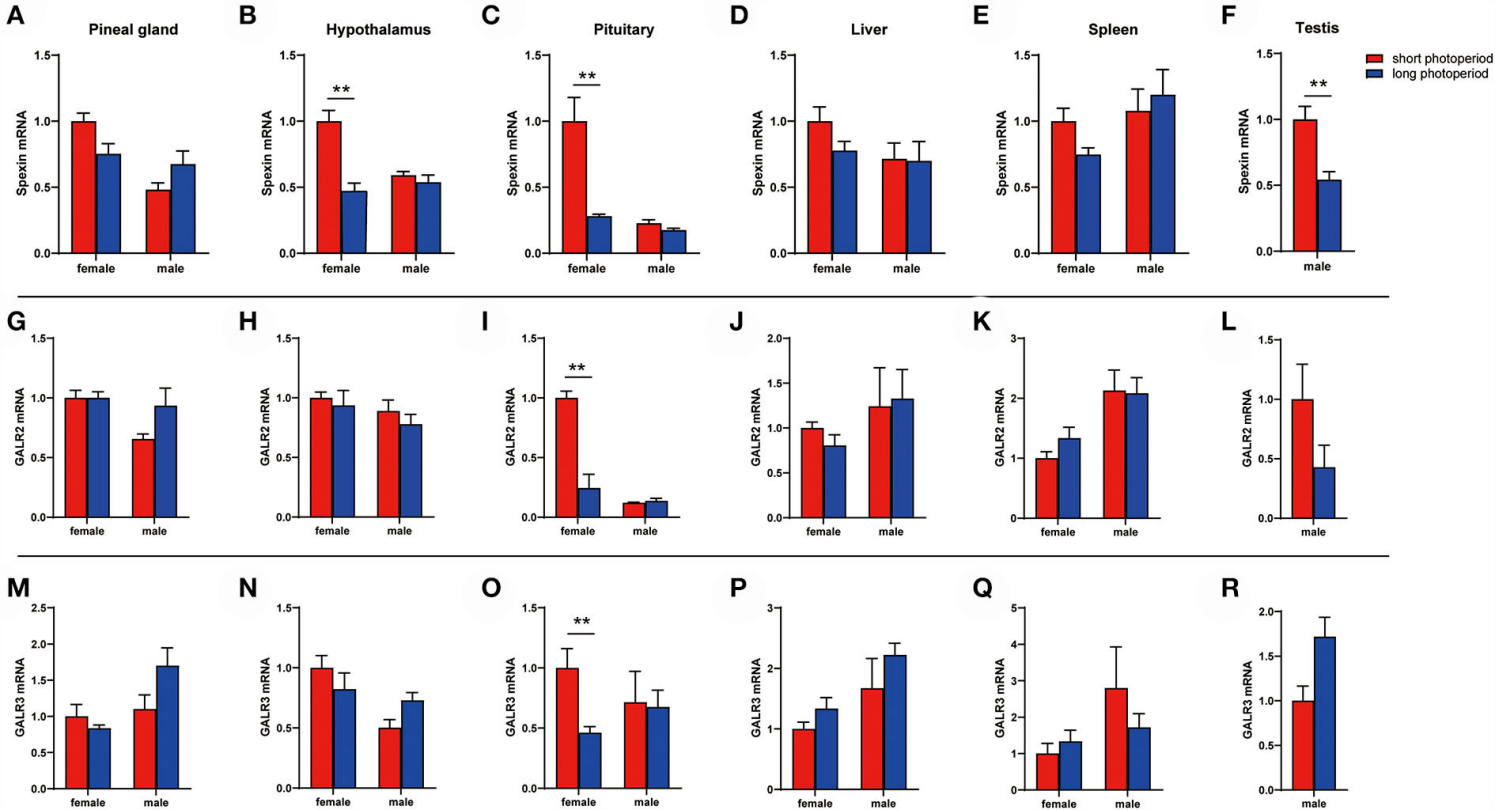
Spexin and GALR2/3 expression during different photoperiods. **(A,G,M)** Gene expression level in pineal gland. **(B,H,N)** Gene expression level in hypothalaus. **(C,I,O)** Gene expression level in pituitary. **(D,J,P)** Gene expression level in liver. **(E,K,Q)** Gene expression level in spleen. **(F,L,R)** Gene expression level in testis. Data are expressed as mean ± SEM (*n* = 3). **P < 0.05.

We found that the long photoperiod inhibited the expression of spexin in the hypothalamus and pituitary (Figures 4B,C), and decreased the mRNA content of GALR2/3 in the pituitary (Figures 4I–O). Moreover, this change was only observed in female geese, and there was no significant difference in the male geese (Figures 4A–R, *P* > 0.05).

### GnRH, GnIH, LHβ, and FSHβ expression during different photoperiods

**Figure 5:**
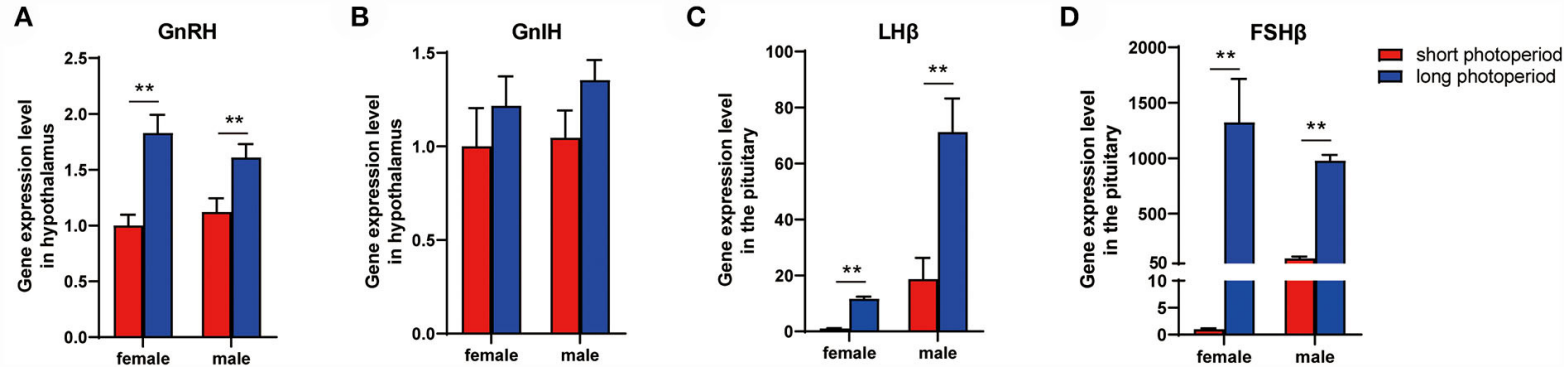
GnRH, GnIH, LHβ, and FSHβ expression during different photoperiods. mRNA level of GnRH **(A)**, GnIH **(B)**, LHβ **(C)**, and FSHβ **(D)** under different photoperiod. Data are expressed as mean ± SEM (*n* = 3). **P < 0.05.

The mRNA levels of gonadotropin-releasing hormone (GnRH), gonadotropin-inhibitory hormone (GnIH), luteinizinghormone (LHβ), and follicle stimulating hormone (FSHβ) were detected using real-time PCR. A significant increase in GnRH, LHβ, and FSHβ levels was observed during the long photoperiod both in female and male (Figure 5).

## Discussion

To date, spexin has been reported in diverse species, including mammals, such as humans (1) and rats (4), bird such as chickens (7), and fish, such as spotted scats (17) and half-smooth tongue soles (18). In this study, we identified the complete nucleotide sequence of spexin cDNA of Yangzhou geese and investigate the expression of spexin and its two receptors (GALR2/3) at the mRNA level in various tissues. Moreover, we examined the effect of spexin in different photoperiods on the reproductive activity of geese.

There are two forms of spexin cDNA in geese, with a short cDNA length of 600-bp and a long cDNA length of 798-bp. Although both nucleic acid sequences can encode the mature peptide of spexin (NWTPQAMLYLKGTQ), compared with the reported spexin sequence (https://www.ncbi.nlm.nih.gov/nuccore/XM_013184566.1), the similarity of the short cDNA was higher, reaching up to 99.75%. Interestingly, through the design of specific primers for the two cDNA sequences, we found that the long cDNA also existed, but the relative expression was very low. Therefore, we performed a follow-up analysis of the short spexin cDNA.

A protein sequence of 117 amino acids was deduced. Previous studies have shown that spexin is highly conserved from fish to mammals (3). Based on the amino acid sequences of spexin from different species, a phylogenetic tree was constructed. Phylogenetic analysis confirmed the close relationship between *Anser cygnoides domesticus* and *A. platyrhynchos*. The spexin of geese is closely related to the spexin sequences of *Gallus gallus, C. japonica, and M. gallopavo*. However, there were obvious species differences from those of mammals and fish.

Spexin is widely distributed in many animal species, based on experiments and genomic analyses (3). Studies on rats (4) and fish (17) have found that spexin mRNA was present in all examined tissues. Currently, only the research by Kolodziejski et al. analyzed spexin mRNA expression levels in birds. The data showed that spexin mRNA was expressed in various tissues, like heart, pancreas, liver, breast muscle, lung, proventriculus, gizzard, kidney, fat, spleen, spinal cord, thigh muscle, duodenum, cecum, and ileum in broiler chicken (7). Therefore, we first analyzed the tissue distribution of spexin and its receptor mRNA in male and female geese. The data showed that spexin, GALR2, and GALR3 were detected in all examined tissues, including the brain, fat, muscle, digestive tract, gonad, gonad, and fat. The two receptors were also expressed in all examined tissues. The wide distribution of spexin and GALR2/3 suggests that spexin-GALR2/3 plays an important role in various physiological activities in geese.

At present, studies on the effects of spexin on reproduction have mostly focused on fish, and no research has been conducted on birds. During ovarian development, the spexin mRNA expression level changes, but there is no effect on GnRH expression after *in vivo* and *in vitro* administration of spexin in orange-spotted grouper and spotted scat (17, 19). In contrast, intraperitoneal injection of the half-smooth tongue sole with spexin significantly decreased GnRH and GnIH mRNA level decreased significantly (18). The uncertainty of the effect of spexin on reproduction is also reflected in the different effects of spexin on the expression of LH and FSH. For example, it has been reported that the plasma levels of LH and FSH decreased when tilapia received spexin by injection (10). Similar phenomena have been observed in goldfish, and the addition of spexin to pituitary slices decreased the firing rate of LH cells (11). These data indicate that spexin acts as a negative factor to suppress the gonadotrope-axis by decreasing the LH and FSH levels in fish. Contrary evidence suggests that the administration of spexin had no effect on LH and FSH in orange-spotted grouper and spotted scat (17, 19). Studies in ewes also support the view that spexin do not affect reproductive activities with unchanged LH and FSH levels (20). Compared with birds, the reproductive activities of teleosts do not depend on a single neuropeptide system; therefore, it is difficult to extrapolate the discovery of teleosts to birds.

Our previous studies have found that different photoperiods affect the reproductive activities of geese. The long-day breeding Yangzhou goose achieves a high reproductive performance by artificially prolonging the light time (9). In this experiment, we found that prolonged light inhibited the expression of spexin in the hypothalamus and pituitary and decreased the mRNA content of GALR2/3 in the pituitary. Because previous studies on fish suggest that spexin may regulate reproductive activities by affecting the gonadotropin axis (10, 11, 18), we measured the mRNA levels of GnRH, GnIH, LHβ, and FSHβ in the hypothalamus and pituitary. The results showed that the expression levels of GnRH, LHβ and FSHβ in the hypothalamus or pituitary increased significantly in the extended light stage, that is, the stage when geese began to lay eggs.

**Figure 6:**
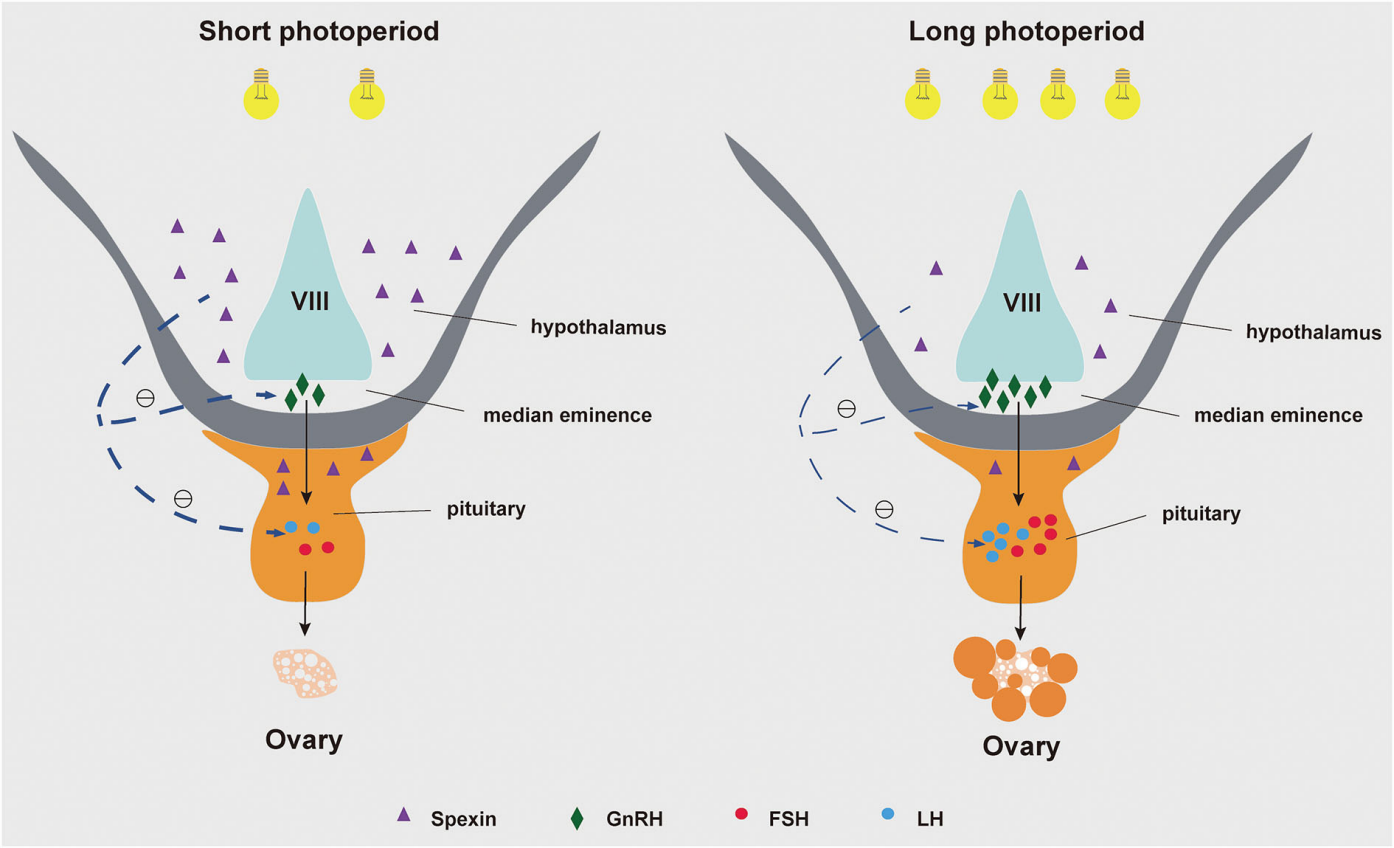
A The role of spexin in goose reproduction during different photoperiods.

In conclusion, prolonged light reducing the spexin and GALR2/3 mRNA content in the hypothalamus and/or pituitary, at the same time GnRH, LHβ, and FSHβ increased, and ultimately activate the gonadotrope-axis of geese. Based on the research on other species, it is speculated that prolonged light exposure may affect GnRH, LHβ and FSHβ mRNA expression by reducing the expression of spexin and its receptor (Figure 6).

## Data availability statement

The original contributions presented in the study are included in the article/supplementary materials, further inquiries can be directed to the corresponding author.

## Ethics statement

The animal study was reviewed and approved by the Research Committee of Jiangsu Academy of Agricultural Sciences.

## Author contributions

JL: conceptualization, methodology, data analysis, and manuscript drafting. SD, ZD, and PY: animal care and sampling. XS: data analysis, manuscript revision, and technical support. CW: animal care and technical support. ML and RC: technical support. HZ: experimental design and supervision. All authors contributed to the article and approved the submitted version.

## Funding

This research was supported by the Jiangsu Agricultural Science and Technology Innovation Fund (CX (20)3148), the National Natural Science Foundation of China (grant number 31972551), and the Key Research and Development Program Project of Anhui Province (202204c06020078).

## Conflict of interest

Authors XS and CW were employed by Anhui Tianzhi-jiao Goose Industry Co., Ltd. The remaining authors declare that the research was conducted in the absence of any commercial or financial relationships that could be construed as a potential conflict of interest.

## Publisher's note

All claims expressed in this article are solely those of the authors and do not necessarily represent those of their affiliated organizations, or those of the publisher, the editors and the reviewers. Any product that may be evaluated in this article, or claim that may be made by its manufacturer, is not guaranteed or endorsed by the publisher.
